# In-depth study of DNA binding of Cys_2_His_2_ finger domains in testis zinc-finger protein

**DOI:** 10.1371/journal.pone.0175051

**Published:** 2017-04-06

**Authors:** Chun-Chi Chou, Shu-Yi Wei, Yuan-Chao Lou, Chinpan Chen

**Affiliations:** Institute of Biomedical Sciences, Academia Sinica, Taipei, Taiwan, ROC; George Washington University, UNITED STATES

## Abstract

Previously, we identified that both fingers 1 and 2 in the three Cys_2_His_2_ zinc-finger domains (TZD) of testis zinc-finger protein specifically bind to its cognate DNA; however, finger 3 is non-sequence–specific. To gain insights into the interaction mechanism, here we further investigated the DNA-binding characteristics of TZD bound to non-specific DNAs and its finger segments bound to cognate DNA. TZD in non-specific DNA binding showed smaller chemical shift perturbations, as expected. However, the direction of shift perturbation, change of DNA imino-proton NMR signal, and dynamics on the ^15^N backbone atom significantly differed between specific and non-specific binding. Using these unique characteristics, we confirmed that the three single-finger segments (TZD_1_, TZD_2_ and TZD_3_) and the two-finger segment (TZD_23_) non-specifically bind to the cognate DNA. In comparison, the other two-finger segment (TZD_12_) binding to the cognate DNA features simultaneous non-specific and semi-specific binding, both slowly exchanged in terms of NMR timescale. The process of TZD binding to the cognate DNA is likely stepwise: initially TZD non-specifically binds to DNA, then fingers 1 and 2 insert cooperatively into the major groove of DNA by semi-specific binding, and finally finger 3 non-specifically binds to DNA, which promotes the specific binding on fingers 1 and 2 and stabilizes the formation of a specific TZD–DNA complex.

## Introduction

DNA-binding proteins initiate gene transcriptional regulation by searching for their target DNA sites among an overwhelming number of non-specific DNA sequences in the nucleus. Theoretically, the protein first binds non-specifically to DNA, then rapidly searches the sequence for the presence of specific binding sites [1]. Therefore, protein–DNA recognition includes at least two steps, non-specific and specific binding [2–4].

Investigating the transient nature of the non-specific protein–DNA binding complex is challenging; however, non-specific binding in the recognition process has received considerable attention [5–11]. Electrostatic interaction is important in dominating non-specific protein–DNA interactions [12,13]. Also, the protein interacting residues can switch roles from a purely electrostatic interaction with the DNA backbone in the non-specific complex to a highly specific binding mode with the base pairs of the cognate sequence [5]. However, a detailed description of the steps that transform the non-specific complex into the specific one in recognition is lacking.

The tandem repeats of classical Cys_2_His_2_ zinc-finger proteins usually function as a transcription factor, with the individual finger domains binding to the cognate DNA (or specific DNA) cooperatively [14–16]. The unbound multiple–zinc-finger protein is elongated and the relative orientation between finger domains is ill-defined because the linker is highly flexible [17]. By contrast, in the zinc-finger protein complexed with the cognate DNA, the linker becomes more rigid and undergoes significant structural changes as compared with the unbound zinc-finger protein [18–21]. Therefore, the binding process in forming a specific zinc-finger protein–DNA complex is not simply a “lock-and-key” mode but is likely stepwise. In addition, base-specific contacts occur from the amino acid side-chains at discrete position -1 and helix positions 2, 3, and 6 in each finger [19]. Also, each finger contributes to the specific binding cooperatively [22], so the number of fingers is an important factor for specificity. Generally speaking, a single zinc-finger protein can only bind to DNA non-specifically [23], and some proteins with two zinc fingers can bind to DNA specifically [24,25].

The crystal structures of a number of proteins complexed with non-specific DNAs have been reported; examples are EcoRV [26], BamHI [27], and lac-repressor [5]. However, the x-ray structure of a modular zinc-finger protein in complex with non-specific DNA is not available. Non-specific DNA binding studies of zinc-finger proteins involved NMR approaches. For example, structural, dynamic, and kinetic investigations of Egr-1 binding to non-cognate DNA shows how Egr-1 efficiently scans DNA and finds its target site rapidly [28]. Moreover, analysis of chemical shift perturbations revealed a similar binding surface of ZNF217 on binding to specific and non-specific DNAs [29]. However, zinc-finger proteins are quite diverse in terms of linkers and tandem repeats. More studies of zinc-finger proteins in complex with specific and non-specific DNAs are needed to gain insight into the interaction mechanism.

**Fig 1:**
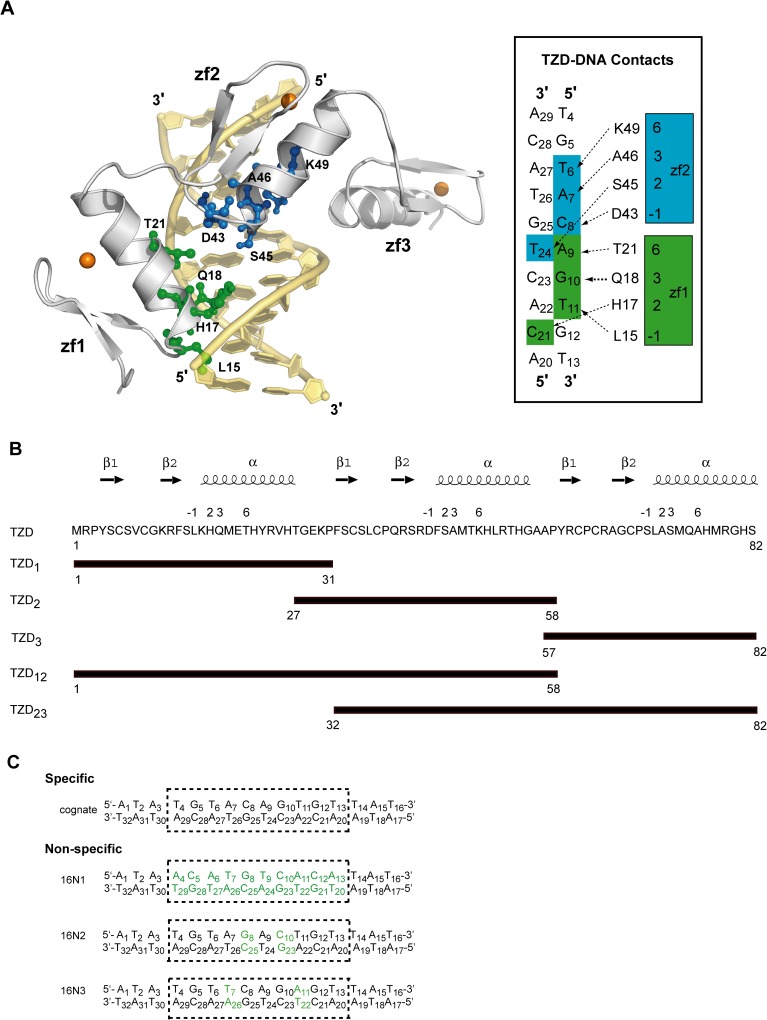
Binding characteristics of the TZD-DNA complex and the design of TZD fragments and non-specific sequences. (**A**) Left, side-chains of the residues at discrete position -1 and helix positions 2, 3 and 6 in zf1 (Leu15, His17, Gln18 and Thr21), shown in green, and zf2 (Asp43, Ser45, Ala46 and Lys49), shown in blue, deeply insert into the major groove of the cognate DNA in TZD-DNA complex model. Zinc ions, Zn2+, are shown in orange balls. Right, schematic of the predicted contacts of zf1 (green) and zf2 (blue) of TZD with the cognate DNA, from positions -1, 2, 3 and 6 of the α-helix. (**B**) Amino acid sequences of TZD in one-letter codes and the range of each finger segment. Secondary structural elements of TZD and positions -1, 2, 3 and 6 of the α-helix that typically make sequence-specific contacts with DNA in classical zinc finger are labeled on the top. (**C**) The cognate (or specific) DNA contains a single 10–bp core region shown in the dashed rectangle. For the three designed non-specific DNAs, 16N1 has the complementary sequence in the core region compared to the cognate DNA. 16N2 and 16N3 have a 2-bp mutation on critical recognition bases, shown in green.

The testis zinc finger protein (TZFP), a promyelocytic leukemia zinc-finger protein (PLZF)-related transcription factor, contains a conserved N-terminal BTB/POZ domain and a C-terminal three-Cys_2_His_2_ zinc-finger domain (TZD). The N-terminal BTB/POZ domain has repressor activity, so TZFP may negatively regulate *Aurora*-C gene expression in spermatocytes [30]. In addition, TZFP is involved in the repression of androgen receptor in mouse testis [31] and a proliferative burst of virus-specific natural killer cells [32]. TZD can specifically bind to the sequence 5’-TGTACAGTGT-3’, located in the upstream flanking sequence of the *Aurora*-C/*Aie1* gene [30]. Previously, we identified that zinc fingers 1 and 2 (zf1, zf2) of TZD specifically bind to the cognate DNA, and finger 3 (zf3) shows non-sequence–specific binding [30,33]. Chemical shift perturbation study for the specific TZD-DNA complex showed that most perturbed residues locate in the N-terminal portions of the α-helices of zf1 and zf2, such as Gln18, Phe44, Ser45 and Ala46, and in the TGEKP linker, such as Thr27, Glu29 and Lys30 [33]. The TZD–DNA docking model revealed that residues Leu15, Lys16, His17, Gln18 and Thr21 in zf1 and Asp43, Phe44, Ser45, Ala46, Lys49 and His50 in zf2 bind to DNA, either by H-bonding or hydrophobic interaction [33]. Interestingly, among these interacting residues, only the amino acid side-chains at discrete position -1 and helix positions 2, 3 and 6 in both zf1 and zf2 deeply insert into the major groove of the cognate DNA, as shown in Fig 1A. By contrast, the perturbed residues in the TGEKP linker are likely due to conformational change in the linker between the free and DNA-bound TZD, such that these residues showed no interaction with DNA. Nevertheless, the role of zf3 in specific recognition remains elusive.

To better understand the recognition process, and further investigate DNA binding characteristics on TZD mainly with NMR techniques, here we constructed three single-finger segments (TZD_1_, TZD_2_ and TZD_3_) and two two-finger TZD segments (TZD_12_ and TZD_23_) (Fig 1B) and designed three non-specific DNAs, with 16N1 having the complementary sequence in the core region compared to the cognate DNA and 16N2 and 16N3 having a 2-bp mutation on critical recognition bases (Fig 1C). From our results, we propose the interaction mechanism of how TZD specifically binds to the cognate DNA.

## Results

### NMR study of non-specific TZD–DNA complexes

**Fig 2:**
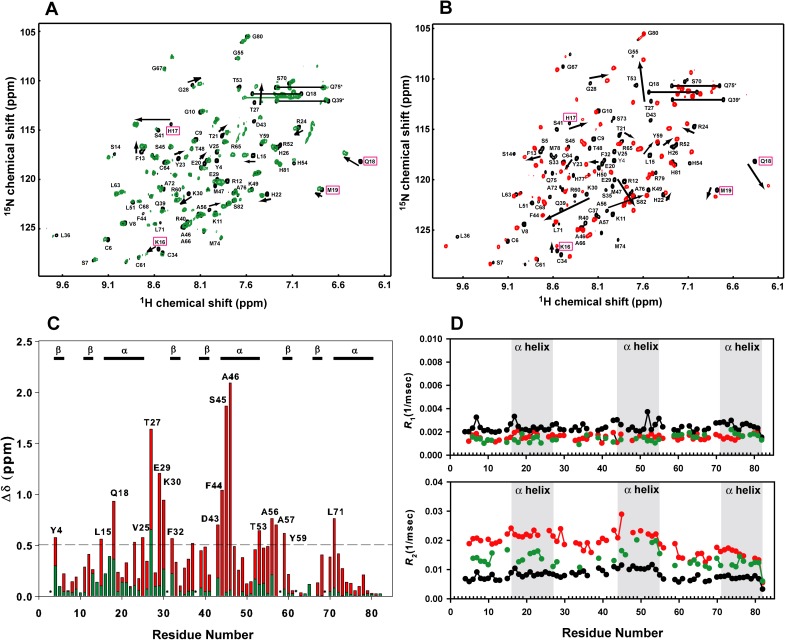
Comparisons of chemical shift perturbations and backbone ^15^N atom dynamics between non-specific and specific TZD–DNA complexes. Superimposition of (**A**) ^15^N-HSQC spectra for the free TZD (black) and TZD bound to the non-specific DNA (green) and (**B**) free TZD (black) and TZD bound to the specific DNA (red). Residues with significant shift changes are shown with black arrows. Compared to non-specific binding in (**A**), shift perturbations with specific binding are larger and the directions of shift perturbations significantly differ, and the represented residues showing chemical shift direction change are in magenta boxes. (**C**) Superimposition of the weighted chemical shift perturbation between non-specific (green) and specific (red) bindings. The secondary structures of TZD are labelled above the histograms and Pro residues are indicated by asterisks. (**D**) Comparison of backbone atom dynamics for ^15^N *R*_1_ and *R*_2_ values among the free TZD (black), non-specific TZD-DNA (green), and specific TZD–DNA (red) complexes.

Three 16-bp DNA duplexes, 16N1, 16N2 and 16N3, were designed for this non-specific TZD-DNA binding study. 16N1 has the complementary sequence of the cognate DNA. 16N2 and 16N3 have a 2-bp mutation on critical recognition bases. 2D ^1^H-^15^N HSQC spectra for the ^15^N-labeled TZD in complex with each non-specific DNA show similar cross-peak patterns (S1 Fig), with the representative spectra shown in Fig 2A. Because of severe overlapping, residues Arg12, Val25, Ser33, Arg42, Phe44, Met47, Ala57, and Ala66 could not be assigned. The spectra significantly differ from those for the free TZD and for TZD bound with specific DNA (Fig 2B), so TZD binding to non-specific DNA should have a different binding mode.

Previously, we found that TZD binding to the cognate DNA causes conformational changes in the linker regions. As well, residues at the N-terminal portions of the α-helix in each finger show significant chemical shift perturbations. In comparison, chemical shift perturbations in the non-specific TZD–DNA complex are much smaller, especially for zf2 and zf3 (Fig 2C). In addition, as shown in S2 Fig, directions of chemical shift change on several residues in the helical region of zf1, such as residues Lys16, His17, Gln18 and Met 19, are opposite between specific and non-specific complexes, which suggests that the binding surfaces between these two binding modes differ. Residues Thr27, Gly28, E29 and Lys30 in linker 1 showed a similar direction and smaller magnitude of shift perturbation as compared with those in the specific TZD–DNA complex, with their cross-peak positions situated between the free and specific complex. Therefore, the linker 1 in non-specific DNA binding adopts a conformational change similar to that in the specific complex.

^15^N relaxation studies showed the unique dynamic properties of TZD bound to non-specific DNA. Because of poor sensitivity of heteronuclear nuclear Overhauser effect (NOE), we show only ^15^N longitudinal relaxation rates (*R*_1_) and ^15^N transverse relaxation rates (*R*_2_) (Fig 2D). The mean *R*_1_ value was 1.42 s^-1^ and mean *R*_2_ value 13.54 s^-1^. The decrease in *R*_1_ value and increase in *R*_2_ value in the non-specific complex reflects the changes in global tumbling between the free and specific complex. Comparison of relaxation data showed that TZD had a lower ^15^N *R*_2_ rate (13.54 < 18.60 s^-1^) in the non-specific than specific complex. The mean *R*_2_/*R*_1_ value was 11.4, 13.6, and 7.39 for zf1, zf2, and zf3, respectively, in the non-specific complex, so zf1 and zf2 have stronger DNA binding than zf3. The free TZD showed enhanced mobility, with a picoseconds to nanoseconds timescale, and became more rigid on binding to non-specific or specific DNA.

### Binding affinities of TZD and finger segments bound to specific and non-specific DNA

**Fig 3:**
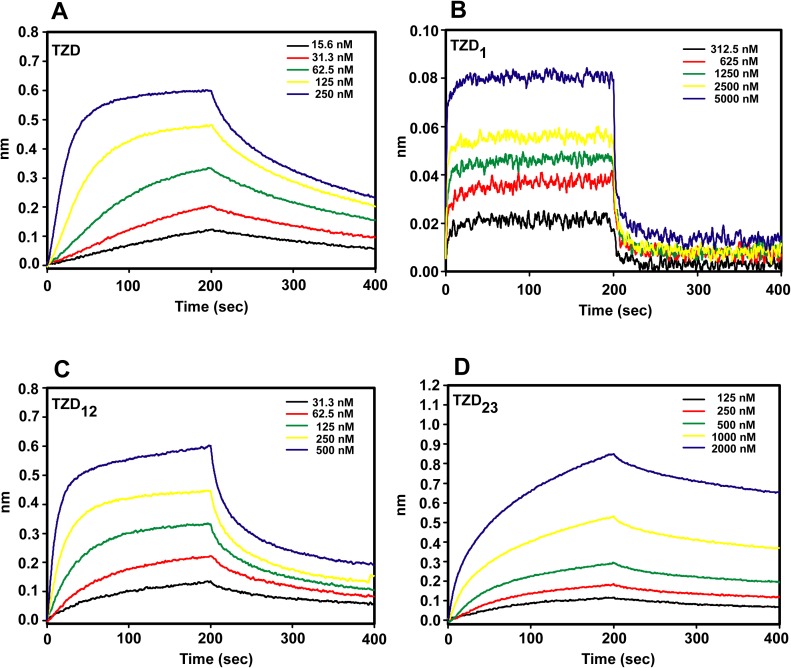
DNA binding kinetics analysis. Spectra of DNA-binding affinities measured by bio-layer interferometry technology (Octet Red system, ForteBio) for TZD and finger segments in complex with the cognate DNA at different concentrations.

DNA-binding affinities of TZD and zinc-finger segments in complex with the specific and non-specific DNA (16N3) were measured by bio-layer interferometry technology (Octet Red system, ForteBio) (Fig 3, Table 1). For single zinc-finger segments bound to specific DNA, the dissociation rate constant was in the 10^−6^ M range for TZD_1_ and could not be determined for TZD_2_ or TZD_3_ because of small responses. The binding affinity was much smaller for TZD_1_ than the specific TZD–DNA complex. This observation agrees with the notion that a single zinc finger of the Cys_2_-His_2_ family is incapable of high-affinity binding to specific DNA. For the two two-finger segments, the dissociation rate constants were in the 10^−7^ M range, whereas TZD_12_ was six-fold stronger than TZD_23_ and ~ 0.5-fold weaker than TZD. This finding agrees well with our previous conclusion that zf1 and zf2 specifically bind to the cognate DNA, but zf3 binding is non-specific. Because we observed distinct DNA binding affinities for these two two-finger segments, the number of zinc fingers is not the only factor determining the binding characteristics on the zinc-finger protein. For the non-specific DNA binding, the dissociation rate constant of TZD-16N3 and TZD_12_-16N3 was 1.44 x 10^−7^ and 5.76 x 10^−7^, respectively. As anticipated, the binding affinity of TZD and TZD_12_ was weaker in non-specific than specific binding (5.65 x 10^−8^ for TZD and 1.05 x 10^−7^ for TZD_12_).

**Table 1:** Dissociation rate constants of TZD and finger segments in complex with specific and non-specific (16N3) DNA.

	TZD_1_	TZD_12_	TZD_23_	TZD
**Specific**				
k_on_ (1/Ms)	7.53(±3.36) × 10^4^	1.33(±0.26) × 10^5^	8.31(±1.18) × 10^3^	1.14(±0.06) × 10^5^
k_off_ (1/s)	1.48(±0.54) × 10^−1^	1.34(±0.13) × 10^−2^	5.59(±0.68) × 10^−3^	6.40(±0.36) × 10^−3^
K_D_ (M)	2.06(±0.58) × 10^−6^	1.05(±0.29) × 10^−7^	6.76(±0.79) × 10^−7^	5.65(±0.35) × 10^−8^
**Non-specific**				
k_on_ (1/Ms)	N/A	6.88(±0.68) × 10^3^	N/A	1.69(±0.02) × 10^5^
k_off_ (1/s)	N/A	3.94(±0.14) × 10^−3^	N/A	2.43(±0.06) × 10^−2^
K_D_ (M)	N/A	5.76(±0.77) × 10^−7^	N/A	1.44(±0.05) × 10^−7^

### Changes in DNA imino-proton NMR signals between specific and non-specific binding

**Fig 4:**
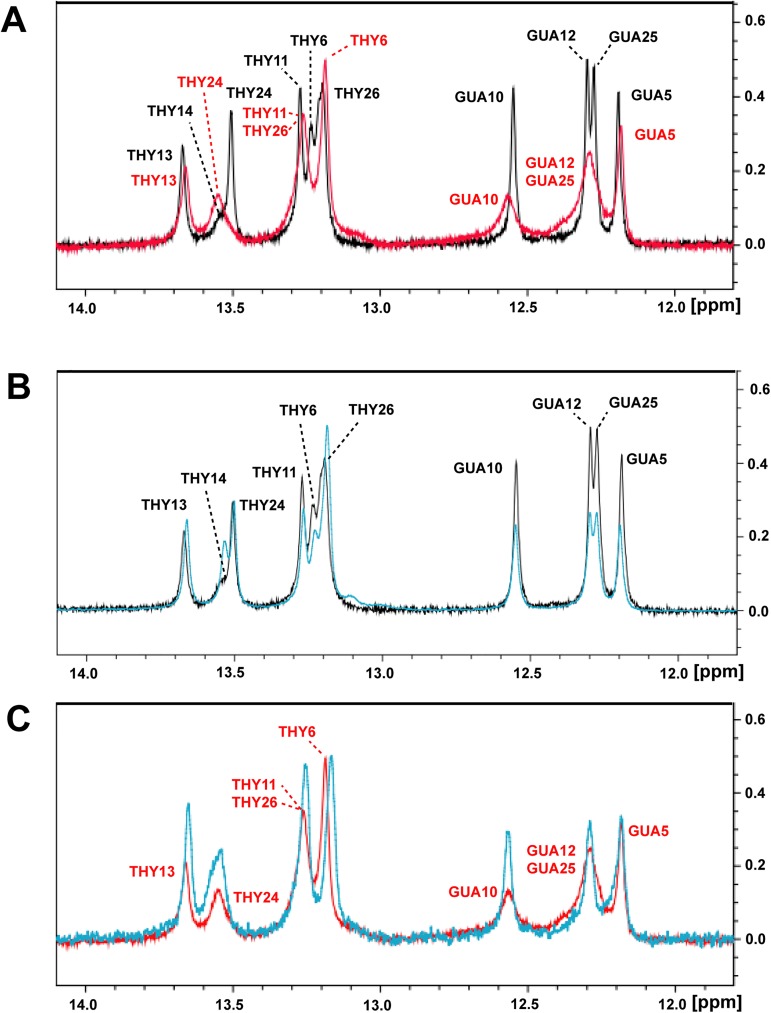
Comparisons of 1D DNA imino-proton NMR signals of the cognate DNA in the free and bound forms. (**A**) Superimposition of imino-proton spectra for the free cognate DNA (in black) and the cognate DNA bound to TZD (in red). Superimposition of imino-proton spectra for (**B**) the free cognate DNA (in black) and the cognate DNA bound to TZD_23_ (in cyan) and (**C**) the cognate DNA in complex with TZD_12_ (in cyan) and TZD (in red).

Changes in DNA imino-proton NMR signals have been widely used to check how protein binds to DNA [34]. With non-specific binding, the protein binds mostly to only the phosphate backbone but not the base of DNA, so the imino-proton signals are highly similar to those of the free DNA. For the specific protein–DNA complex, the imino-proton spectra significantly differ from that for the free DNA because of the interaction between the DNA base and protein. We adopted this strategy for analyzing TZD and its finger segments. 1D imino-proton NMR spectra for the cognate DNA superimposed on that of the specific TZD–DNA complex (Fig 4A) showed significant chemical shift changes for imino protons of THY6, THY24 and THY26. For the single zinc-finger segment bound to specific DNA, the DNA imino-proton spectra are similar to that for the free DNA (data not shown), which suggests that a single zinc-finger protein bound to cognate DNA is non-specific or does not bind at all. For the two–zinc-finger segments, the imino-proton spectra for the TZD_23_–DNA complex were also very similar to that for the free DNA (Fig 4B), so the binding of TZD_23_ with the cognate DNA was non-specific. As anticipated, the imino-proton NMR spectra for TZD-DNA and TZD_12_-DNA were very similar (Fig 4C) because zf1 and zf2 of TZD both play a role in sequence-specific binding, as previously mentioned. This observation further confirms that the overall binding mode of TZD_12_ bound to the cognate DNA seems to be specific, as for TZD.

### DNA recognition of single zinc-finger segments with the cognate DNA

**Fig 5:**
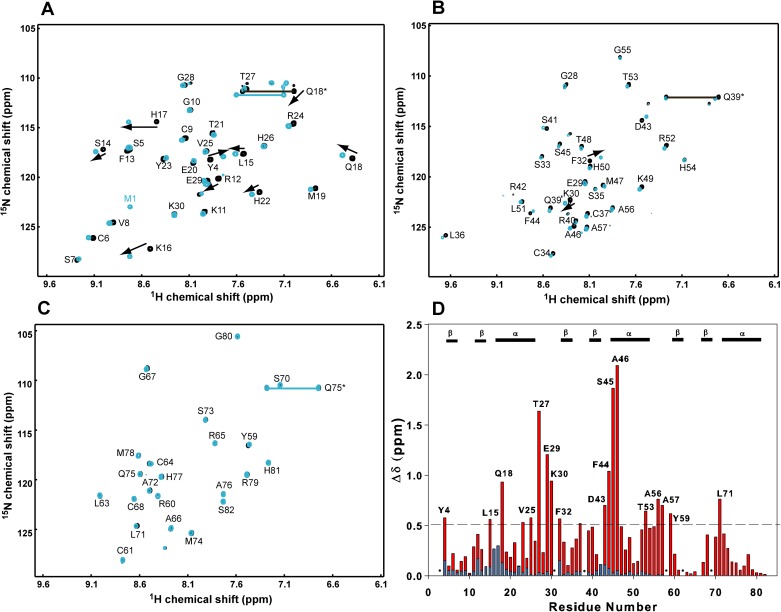
Comparisons of chemical shift perturbations of single-finger segments. Superimposition of ^1^H-^15^N HSQC spectra for (**A**) free TZD_1_ (in black) and TZD_1_ bound to the cognate DNA (in blue) with assignments annotated on the free TZD_1_, (**B**) free TZD_2_ (in black) and TZD_2_ (in blue) bound to the cognate DNA with assignments annotated on the free TZD_2_ and (**C**) free TZD_3_ and TZD_3_ bound to the cognate DNA with assignments annotated on the free TZD_3_ (both spectra are nearly identical). Residues with significant shift changes are shown with black arrows. (**D**) Superimposition of the weighted chemical shift perturbations for the specific TZD–DNA complex (red) and the single-finger segment bound to the cognate DNA (blue). The secondary structures of TZD are labelled above the histograms and Pro residues are indicated by asterisks.

We concluded in our previous study that DNA specific and non-specific binding occur simultaneously in the TZD–DNA complex. In this work, we investigated the chemical shift perturbations of single zinc-finger segments bound to the cognate DNA (Fig 5). Both TZD_1_ and TZD_2_ weakly and non-specifically bound to cognate DNA because we observed very small shift perturbations and the shift perturbation direction was similar to that for the non-specific TZD–DNA complex (Fig 5D). In comparison, the chemical shift perturbation of TZD_3_ is barely seen, even by adding excessive DNA, which suggests no interaction between TZD_3_ and cognate DNA. Thus, the DNA binding mode greatly differs between the single zinc finger and TZD bound to cognate DNA. Because the high-affinity DNA binding is not seen in the single finger bound to cognate DNA, the interaction between the single finger and cognate DNA should be non-specific, and as compared with the specific TZD–DNA complex, the corresponding chemical shift perturbation is much smaller.

### DNA recognition of two–zinc-finger segments with the cognate DNA

**Fig 6:**
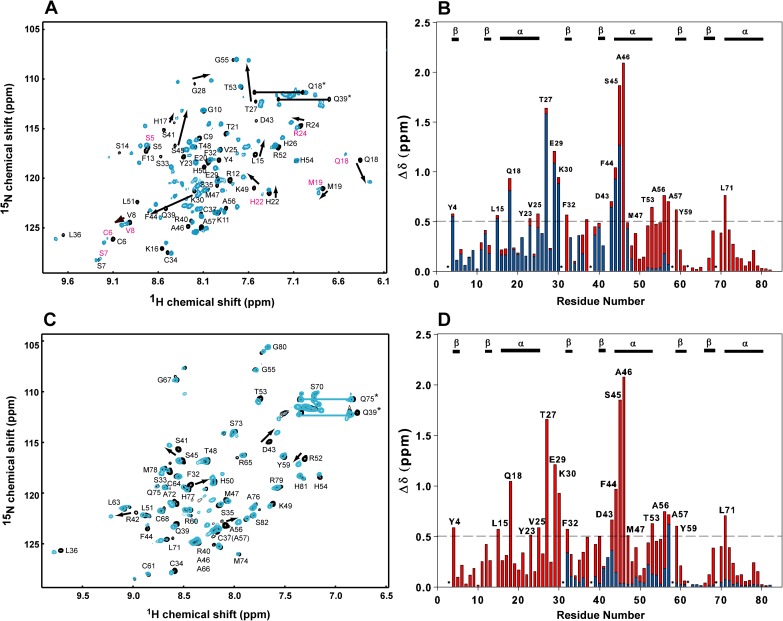
Comparisons of chemical shift perturbations of two-finger segments. Superimposition of (**A**) ^1^H-^15^N HSQC spectra for the free TZD_12_ (in black) and TZD_12_ bound to the cognate DNA (in blue) with assignments annotated on the free TZD_12_, (**B**) weighted chemical shift perturbations for the specific TZD–DNA complex (red) and TZD_12_ bound to the cognate DNA (blue), (**C**) ^1^H-^15^N HSQC spectra for the free TZD_23_ (in black) and TZD_23_ bound to the cognate DNA (in blue) with assignments annotated on the free TZD_23_, and (**D**) weighted chemical shift perturbations for the specific TZD–DNA complex (red) and TZD_23_ bound to the cognate DNA (blue). Residues with significant shift changes are shown with black arrows. The secondary structures of TZD are labelled above the histograms and Pro residues are indicated by asterisks.

We also studied DNA binding with TZD_12_ and TZD_23_ to examine their DNA binding characteristics. TZD_12_ in complex with the cognate DNA showed larger chemical shift perturbations as compared with the single zinc finger (Fig 6A). Because of severe overlapping on some cross peaks, we could only assign 47 of 52 residues in the HSQC spectrum. Several residues in zf1, such as Ser5, Ser7, Val8, Gln18, Met19, His22 and Arg24, contained two sets of resonances with distinct intensities. The major ones with stronger intensities are superimposed on those of TZD bound to cognate DNA, but the minor ones are well superimposed on those of TZD bound to non-specific DNA. Therefore, on binding to cognate DNA, the zf1 of TZD_12_ features both specific and non-specific binding modes, which are slowly exchanged in the NMR timescale. Furthermore, the TGEKP linker in both TZD and TZD_12_ have the same shift perturbation patterns, so TZD_12_ likely binds to cognate DNA specifically (Fig 6B). Nevertheless, we observed smaller chemical shift perturbations in the helix region of zf2, such as residues Asp43 and Ser45, and cross peaks from Arg52 to Ala57 were nicely superimposed with those of TZD in non-specific binding, so the binding characteristics of zf2 of TZD_12_ differ from that of zf2 of TZD in specific and non-specific binding. Therefore, TZD_12_ in complex with DNA presents a state between the specific and non-specific binding. With the result that the binding affinity of the TZD_12_–DNA complex is ~0.5-fold that of the TZD–DNA complex, we named the binding mode of TZD_12_ in complex with cognate DNA “semi-specific binding”. By contrast, the minor ones with weaker intensities undoubtedly bind to DNA in a non-specific mode because they have highly similar shift perturbations as for those with non-specific binding. We concluded that TZD_12_ binding to cognate DNA represents mostly semi-specific binding, with small amounts showing non-specific binding, both of which exist simultaneously and switch slowly in the NMR timescale.

For TZD_23_ bound to the cognate DNA, we found chemical shift patterns similar to those for the free TZD (Fig 6C) and much smaller chemical shift perturbations (Fig 6D), especially for zf2, so DNA binding on zf2 differs from the specific binding seen in the TZD–DNA complex. Thus, TZD_23_ bound to cognate DNA should feature only non-specific binding.

**Fig 7:**
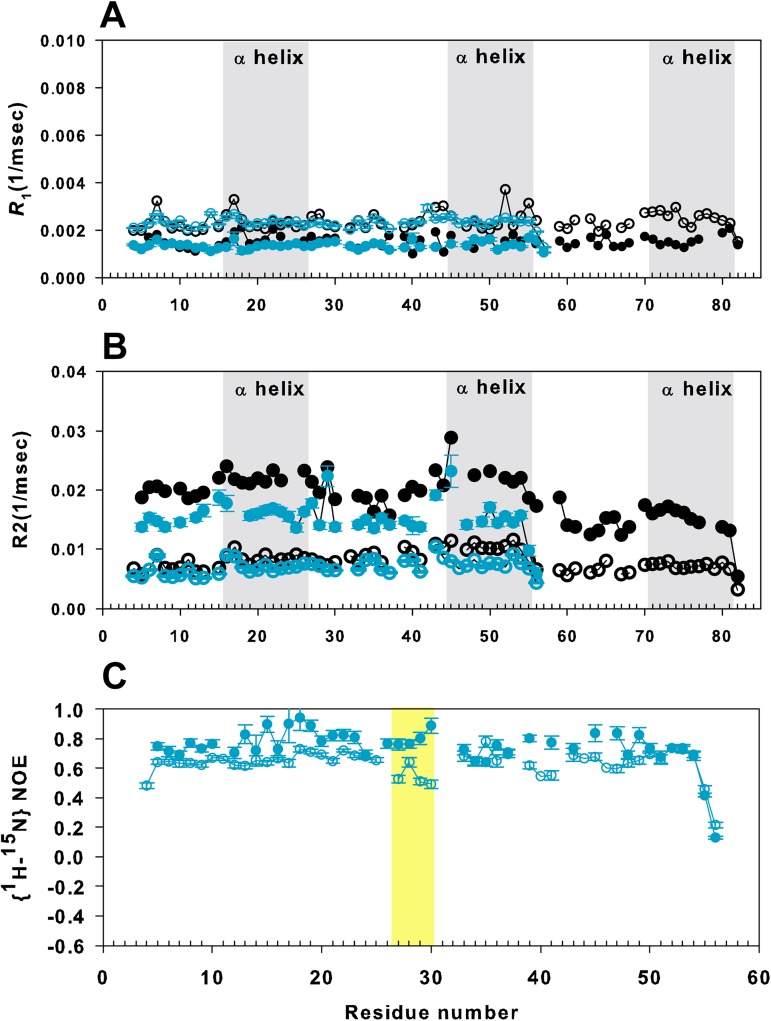
^15^N spin relaxation measurements for TZD_12_. (**A**),(**B**) Comparison of ^15^N *R*_1_ and *R*_2_ relaxation data among free TZD_12_ (blue unfilled circles), TZD_12_ bound to the cognate DNA (blue filled circles), free TZD (black open circles) and TZD in specific binding (black filled circles). To clarify, the helical regions are shown with grey background. (**C**) Comparison of ^1^H-^15^N heteronuclear nuclear Overhauser effect of TZD_12_ in the free (blue unfilled circles) and bound (blue filled circles) forms. The TGEKP linker shown in yellow background between fingers 1 and 2 clearly becomes more rigid when bound to the cognate DNA. Error bars represent fitting errors.

We analyzed the dynamic properties of TZD_12_-DNA and TZD_23_-DNA by ^15^N relaxation studies to investigate how the dynamics adjusts during protein–DNA recognition. In the semi-specific TZD_12_–DNA complex, the mean *R*_1_ value was 1.34 ± 0.14 and mean *R*_2_ value 14.80 ± 3.78 s^-1^. The mean *R*_2_/*R*_1_ values were 12.76 and 10.95 for zf1 and zf2, respectively, which correspond to τ_c_ values of 10.63 and 9.94 ns. The mean *R*_1_ values did not differ between semi-specific and specific binding modes (Fig 7A), and the *R*_2_ value was between those for the free and specific complexes (Fig 7B). ^1^H-^15^N NOE data showed that the TGEKP linker became rigid, similar to that with specific binding (Fig 7C). In comparison, the mean *R*_2_ value for TZD_23_ was between that for the free and specific complexes, with smaller magnitude, 12.04 ± 3.99 s^-1^ and 8.73 ± 1.76 s^-1^ for zf2 and zf3, respectively. However, the resulting mean R_2_ for zf3 was similar to that for the free TZD.

We conclude that the binding mode for the TZD_12_–DNA complex is semi-specific. In contrast to TZD_12_, on binding to the cognate DNA, TZD_23_ exhibited less increase in ^15^N *R*_2_ rate, chemical shift perturbation and binding affinity, which implies a non-specific binding mode.

## Discussion

DNA recognition by classical Cys_2_His_2_ zinc-finger proteins is essential for specific regulation of genes. Most studies and comparisons have mainly focused on the free protein as well as the specific protein–DNA complex in examining DNA recognition. Non-specific protein–DNA binding is an important intermediate step in the recognition process, so in this work we present evidence to show that TZD binding to the cognate DNA is stepwise, initially non-specific, then semi-specific, and finally specific.

### Specific and non-specific binding surfaces are not the same

Recently, several studies reported the characteristics of non-specific binding on multiple zinc fingers. For example, Egr-1, containing three zinc finger domains, has smaller chemical shift perturbations with non-specific than specific binding. The DNA binding surface of ZNF217_F67, containing two zinc finger domains, is very similar with specific and non-specific binding, because changes in chemical shift perturbations are in the same direction. In this study, we found smaller chemical shift perturbations for each zinc finger in the non-specific TZD–DNA complex, which is consistent with Egr-1[28] and ZNF217_F67[29]. However, in TZD, we also found that several perturbed residues actually have opposite directions for specific and non-specific binding, which is different from ZNF217_F67. With this unique observation, we concluded that the binding surface for TZD is not the same with the specific and non-specific complexes. Therefore, the binding surface of tandem-repeat zinc fingers in non-specific and specific binding is not always the same.

### Binding preference of zf1 in the specific TZD–DNA complex

NMR study revealed the asymmetrical roles of multiple–zinc-finger proteins when Egr-1 diffuses on non-specific DNA [28]. Generally speaking, the binding specificity and affinity in the complex of multiple–zinc-finger protein and DNA are determined cooperatively. A single–zinc-finger peptide, such as Xfin-31 [23], non-specifically binds to DNA, although the single Cys_2_His_2_ zinc-finger domain of the GAGA protein flanked by basic residues shows high-affinity specific DNA binding [35]. For two–zinc-finger proteins, Tramtrack [24] and ZNF217 [25] show sequence-specific DNA binding. In this study, among the three single-finger segments of TZD, TZD_1_ non-specifically bound to cognate DNA and had the strongest binding affinity. Compared to TZD_23_ in complex with DNA in non-specific binding, the mode of TZD_12_ binding to the cognate DNA was between the specific and non-specific binding. zf1 rather than zf3 cooperated with zf2 to form a semi-specific complex similar to TZD, especially in zf1 and the TGEKP linker, because their chemical shift perturbations were very close to those for the TZD–DNA complex. We concluded that zf1 has the binding preference in cognate DNA recognition because it has stronger binding affinity to DNA and plays important roles in non-specific, semi-specific and specific bindings.

### Switch between variant binding modes

Comparison of the *lac* DNA binding domain in complex with the cognate and non-specific DNA revealed that the same set of residues can switch roles from a purely electrostatic interaction in the non-specific complex to a highly specific binding interaction with the base pairs of the cognate DNA [5]. Also, the binding process of forming the complex of multiple–zinc-finger protein and DNA is not a “lock-and-key” mode because of conformation changes in the linkers and orientation changes from the free to bound zinc fingers: it is more likely stepwise. However, the transient nature of the non-specific complex represents many difficulties in observing the existence of non-specific binding when the DNA-binding protein binds to the cognate DNA. As a result, the binding process of a multiple–zinc-finger protein bound to the cognate DNA is still elusive.

**Fig 8:**
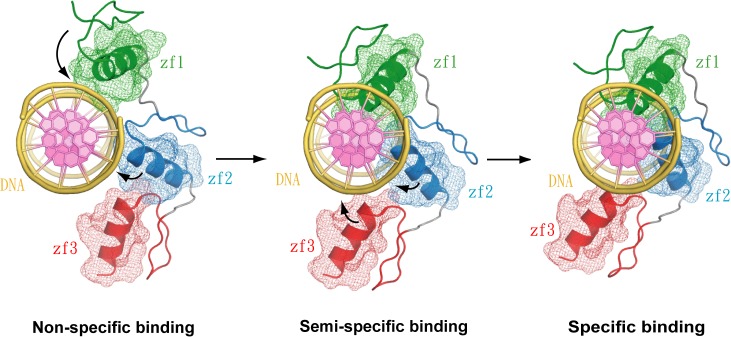
The proposed sequential binding scheme of TZD bound to the cognate DNA. In the TZD–DNA complex, the cognate DNA is represented by a cartoon, with the phosphate backbone in yellow and base in violet. TZD is also represented by a cartoon, with the helix and surface mesh of zinc fingers 1, 2 and 3 shown in green, blue and red, respectively; the β-sheet in each finger is omitted for clarity. Initially, TZD interacts with the cognate DNA in non-specific binding, then fingers 1 and 2 switch from non-specific binding to semi-specific binding, with finger 1 almost fitting into the major groove completely but finger 2 not fitting. Finally, the semi-specific binding is improved by the non-specific binding on finger 3 and the DNA binding is then transformed to the specific mode, with fingers 1 and 2 fitting into the major groove completely, but finger 3 binding non-specifically to DNA.

In this study, we found that TZD_12_ binding to the cognate DNA showed two sets of NMR resonances in zf1, corresponding to semi-specific and non-specific binding, which indicates that the switch occurs between non-specific and semi-specific binding rather than between free and semi-specific binding. Accordingly, TZD_12_ bound to the cognate DNA is non-specific initially, then switches to semi-specific binding. Also, we found that TZD_1_ has stronger binding affinity than TZD_2_ and TZD_3_, although the binding is non-specific. Bio-layer interferometry experiments indicate that the association rate is much faster for TZD_12_ than TZD_23_ (1.33 x 10^5^ vs 8.31 x 10^3^ k_on_). Therefore, zf1 and zf2 of TZD likely have binding preference when binding to cognate DNA. zf1 and the TGEKP linker of TZD_12_ specifically binds to cognate DNA, and TZD_23_ can only bind to cognate DNA non-specifically; however, zf1 alone can only bind to cognate DNA non-specifically. Thus, for specific binding, zf1 and zf2 of TZD seem to cooperatively bind to cognate DNA first. With all these data, we propose the binding scheme of TZD in complex with cognate DNA (Fig 8). In the beginning, TZD may interact with the phosphate backbone of DNA non-specifically, then zf1 and zf2 cooperatively switch to semi-specific binding and insert into the major groove of DNA, and finally zf3 non-specifically binds to DNA, which promotes the specific binding on zf1 and zf2, especially zf2. Therefore, a specific TZD–DNA complex is formed synergistically. The non-sequence specific binding for zf3 plays an important role in the formation of a specific complex.

In this work, we defined a semi-specific binding based on DNA binding characteristics of TZD_12_ bound to cognate DNA. To our knowledge, this kind of binding has not been reported for zinc-finger proteins. Furthermore, we confirmed that zf3 has much weaker binding affinity than both zf1 and zf2 and can only bind to DNA non-specifically. However, such weak binding is necessary for the formation of a specific TZD–DNA complex.

## Materials and methods

### Sample preparation

The DNA fragments encoding the single zinc finger domain of mouse TZD, TZD_1_ (TZFP_348-377_), TZD_2_ (TZFP_373-404_) and TZD_3_ (TZFP_403-428_), were cloned into a modified pET32a (Novagen) vector that was inserted a Tobacco Etch Virus (TEV) protease-cleavable site (Glu-Asn-Leu-Tyr-Phe-Gln-Gly) at the N-terminus. The DNA fragment encoding two finger domains, TZD_12_ (TZFP_348-404_) and TZD_23_ (TZFP_378-428_), were cloned into a modified pET28a (Novagen) vector that was inserted a TEV protease-cleavable site before the target protein. All proteins were expressed in *Escherichia coli* BL21 (DE3). To prepare isotopically labeled (^15^N and ^15^N/^13^C) TZD_1_, TZD_2_ and TZD_3_, cells were grown in M9 minimal medium with extra 50 μM ZnCl_2_ containing ^15^NH_4_Cl (1 g/l) and/or ^13^C-glucose (2 g/l) at 37°C. Cells were induced with 0.5 mM IPTG for an additional 15 h at 18°C until OD_600_ reached 0.8. Cells were then lysed by microfluidizer, and proteins were purified by nickel–nitrilotriacetic acid (Ni–NTA) affinity chromatography. The thioredoxin with His_6_-tag and target protein were separated by cleavage of TEV protease. Target proteins were purified again by successive ion exchange chromatography. Purity and authenticity of the recombinant proteins were verified by SDS–PAGE and mass analysis. Finally, target proteins were dialyzed and concentrated with buffer (50 mM acetic acid and 20 mM NaCl) at pH 6.0 for NMR study. For TZD_12_ and TZD_23_, cells were grown in MOPS minimal medial containing ^15^NH_4_Cl (1 g/l) and/or ^13^C-glucose (2 g/l) at 37°C. Cells were induced with 1 mM IPTG for additional 3 h at 37°C until OD_600_ reached 0.6. The harvested cells were resuspended in buffer (50 mM Tris-HCl, pH 8.0, 1 mM EDTA, and 30 mM NaCl) and disrupted by microfludizer. Inclusion bodies were collected by centrifugation and protein was extracted with 8 M urea containing 30 mM β-mercaptoethanol. The denaturant was purified by nickel–nitrilotriacetic acid (Ni–NTA) affinity chromatography and exchanged by dialysis with solutions containing 50 mM acetic acid and 0.2 mM dithiothreitol (DTT). The His_6_-tag and target protein were separated by cleavage of TEV protease. After centrifugation, the supernatant was loaded on CM52 column equilibrated with buffer (50 mM acetic acid and 0.2 mM DTT) and eluted by a gradient of NaCl. The target proteins were purified again by gel-filtration chromatography. The eluted fractions containing protein were titrated and dialyzed against buffer (50 mM acetic acid and 6 mM ZnCl_2_). The single-stranded DNAs were purchased from MDBio Inc. (Taiwan) and the double-stranded DNA was prepared by mixing equal amounts of two complementary deoxynucleotides, heating to 85°C for 10 min and cooling slowly to room temperature.

### DNA binding kinetics analysis

All DNA binding assays were performed in 96-well microplates at a volume of 200 μl per well at 30°C by using the Octet Red system (FortéBio, Inc., Menlo Park, CA). The 3’-biotinylated 16-bp dsDNA dissolved in the buffer (50 mM glacial acetic acid, 20 mM NaCl and 0.5 mM NaN_3_, pH6 with 0.01% Tween-20) with a concentration of 1 μg/ml was immobilized to the streptavidin biosensor tips (FortéBio, Inc.) for ~60 s. The immobilized response was about 0.07–0.2 nm. After reaching baseline in the running buffer (50 mM glacial acetic acid, 50 mM NaCl and 0.5 mM NaN_3_, pH6 with extra 0.01% Tween-20, 0.5 mg/ml BSA and 0.5 mM DTT) for 120 s, all sensors with or without biotinylated dsDNA were dipped into different concentrations of proteins or the blank buffer (as sample reference) for 200-s association and then moved into the running buffer for 200-s dissociation. Data were analysed by a double reference subtraction method (sample and sensor references) with the Octet Red analysis software. Kinetic parameters were evaluated by using a 1:1 binding model with global fitting of three independent experiments.

### NMR experiments

NMR experiments were performed at 25°C on Bruker AVANCE 600 or AVANCE 800 spectrometers equipped with a cryo-probe. Each sample contained about 0.25 ml of ~1 mM protein in a Shigemi NMR tube. The NMR spectra were obtained as described previously. For backbone resonance assignment, 3D triple-resonance experiments, HN(CO)CA, HNCA, CBCA(CO)NH and HNCACB were performed in H_2_O. All NMR spectra were processed by using the Bruker XWINNMR and NMRPipe package [36], and analyzed by using NMRView [37]. The weighted chemical shift perturbations for backbone ^15^N and ^1^H^N^ resonances were calculated as follows: Δδ = [(Δδ_HN_)^2^ + (Δδ_N_/5)^2^]^0.5^. NMR spin–relaxation experiments were carried out at 298K. For the free proteins, the relaxation delays in the *T*_1_ experiment were 10, 20, 30, 40, 60, 90, 150, 300, 600, 1000 and 1500 ms, and those in the *T*_2_ experiments were 17.15, 34.30, 51.45, 68.60, 85.76, 102.91,120.06, 137.21, 154.43, 188.67, and 222.97 ms. For the bound proteins, the relaxation delays in the *T*_1_ experiment were 10, 20, 30, 40, 60, 90, 150, 300, 600, 1000 and 1500 ms, and those in the *T*_2_ experiments were 17.15, 34.30, 51.45, 68.60, 85.76, 102.91,120.06, 137.21, 154.43, 188.67, and 222.97 ms. ^1^H-^15^N steady-state hNOE values were measured by recording spectra with or without a ^1^H saturation period of 3 s. To determine the *R*_1_ and *R*_2_ relaxation rate, resonance intensities were extracted and fitted to a non-linear, least-squares curve as a function of the relaxation delay time by using the L-M non-linear fitting routines in NMRView.

## Supporting information

S1 Fig ^15^N-HSQC spectra of TZD for non-specific complexes. (DOCX)

S2 Fig Observation of distinct directions on chemical shift perturbations between specific and non-specific complexes (DOCX)
