# Supplementary material for: In-depth study of DNA binding of Cys2His2 finger domains in testis zinc-finger protein
Source: PLoS One. 2017 Apr 6;12(4):e0175051. doi: 10.1371/journal.pone.0175051 (PMC5383199; doi:10.1371/journal.pone.0175051)
Supplement: S1 Fig — (DOCX) [file pone.0175051.s001.docx]

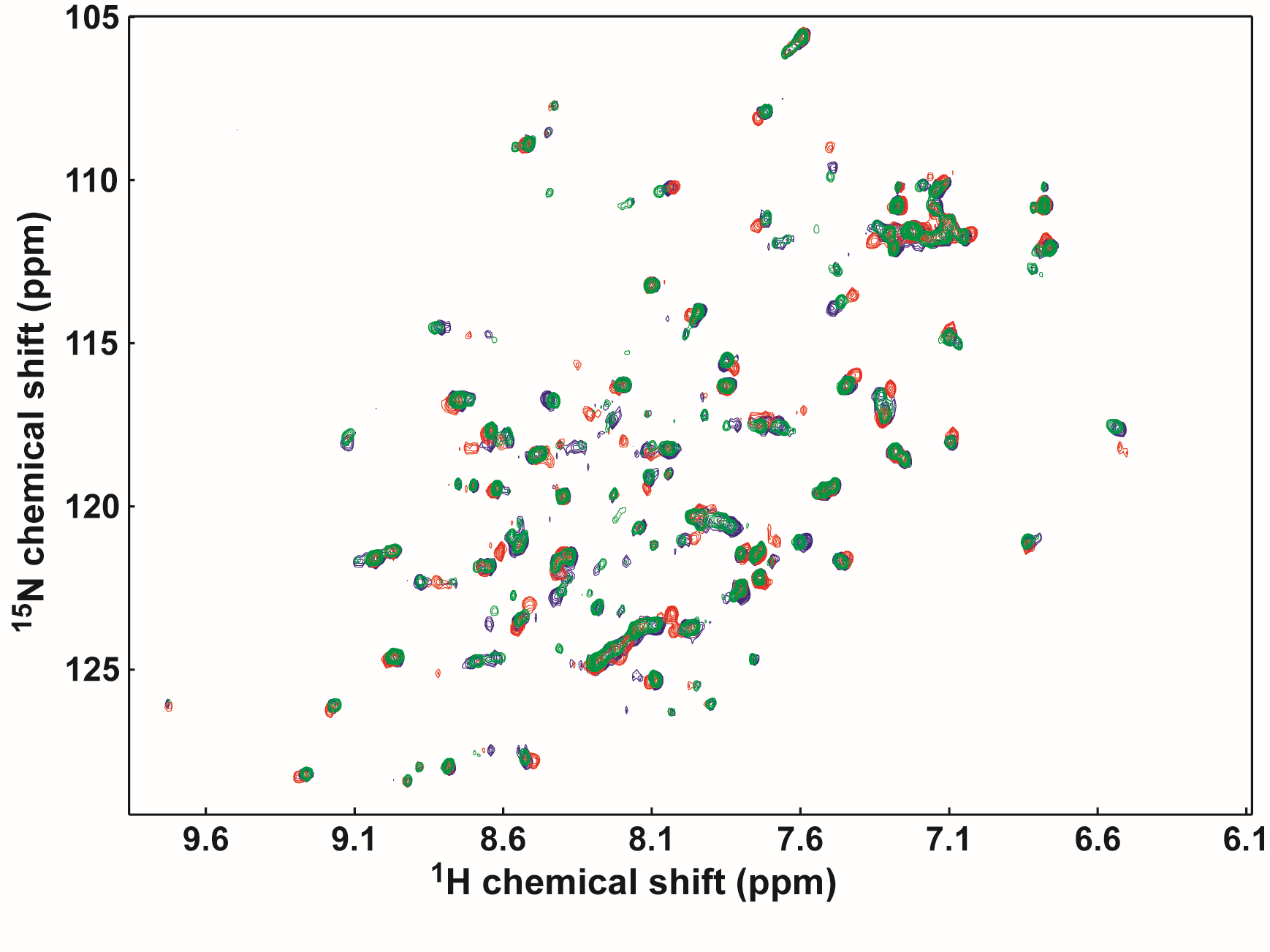


**S1 Fig. ^15^N-HSQC spectra of TZD for non-specific complexes.**

Overlay of ^15^N-HSQC spectra of TZD in complex with the non-specific DNA sequences, 16N1, 16N2 and 16N3, shown in blue, red and green, respectively, illustrating similar patterns.
