# Supplementary material for: In-depth study of DNA binding of Cys2His2 finger domains in testis zinc-finger protein
Source: PLoS One. 2017 Apr 6;12(4):e0175051. doi: 10.1371/journal.pone.0175051 (PMC5383199; doi:10.1371/journal.pone.0175051)
Supplement: S2 Fig — (DOCX) [file pone.0175051.s002.docx]

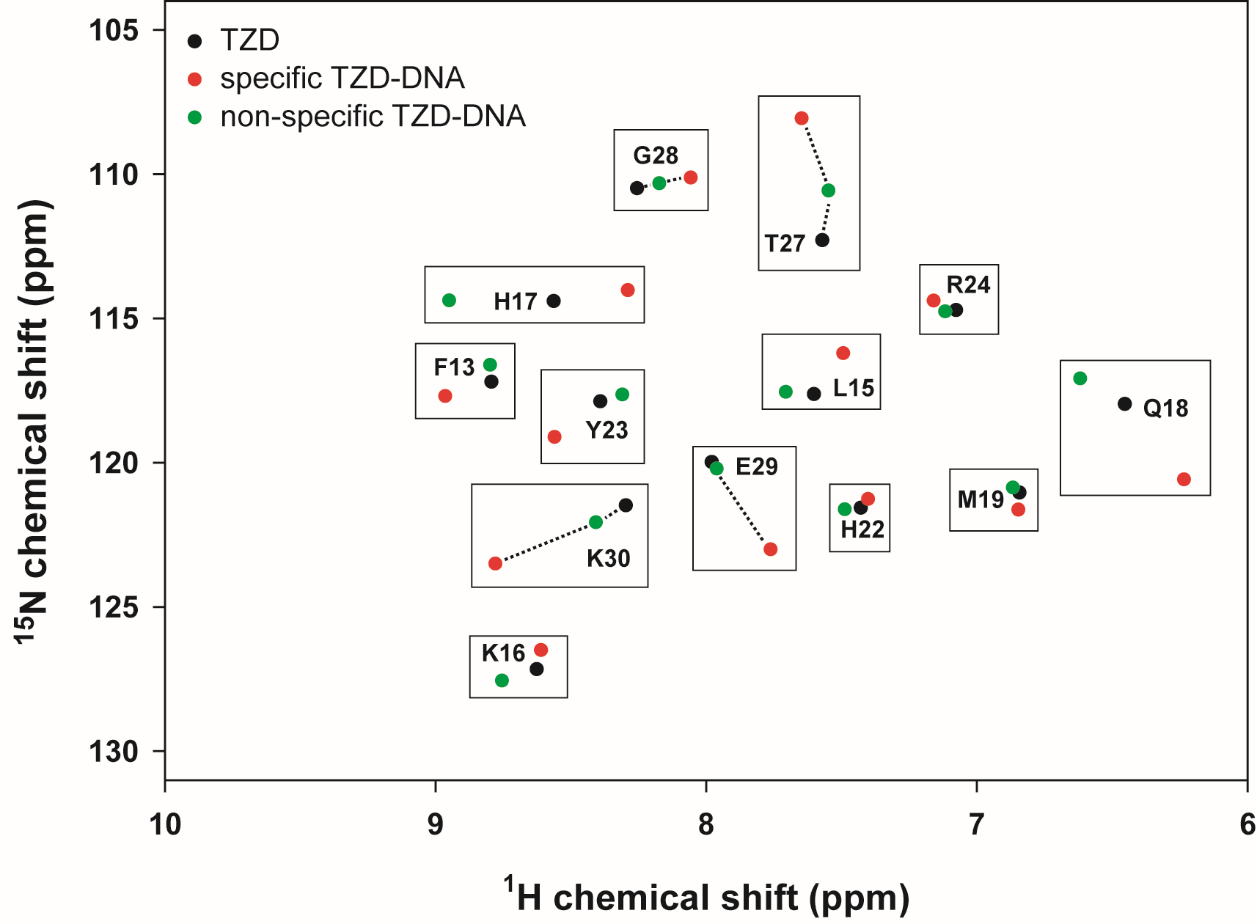


**S2 Fig. Observation of distinct directions on chemical shift perturbations between specific and non-specific complexes**

To clarify, chemical shift changes of some particular residues observed in 2D ^1^H-^15^N HSQC spectra among the free TZD (in black), TZD bound to specific (in red) and non-specific (in green) DNAs are drawn. Residues in TGEKP linker residues are labelled with dotted lines.
